# Effects of immune suppression for transplantation on inflammatory colorectal cancer progression

**DOI:** 10.1038/s41389-018-0055-5

**Published:** 2018-06-19

**Authors:** Imerio Angriman, Lucrezia Furian, Melania Scarpa, Matteo Fassan, Susan Morgan, Andrea Porzionato, Andromachi Kotsafti, Luca Saadeh, Cristina Silvestre, Raffaele De Caro, Amedeo Carraro, Umberto Tedeschi, Romeo Bardini, Paolo Rigotti, Massimo Rugge, Carlo Castoro, Ignazio Castagliuolo, Marco Scarpa

**Affiliations:** 1 General Surgery Unit, University Hospital of Padua, Padua, Italy; 2 Kidney Transplant Unit, University Hospital of Padua, Padua, Italy; 3 Esophageal and Digestive Tract Surgical Unit, Veneto Institute of Oncology IOV-IRCCS, Padua, Italy; 4 Surgical Pathology Unit, Department of Medicine DIMED, University Hospital of Padua, Padua, Italy; 5 Pathology Unit, Sheffield Teaching Hospitals, Sheffield, UK; 6 Department of Neurosciences, University Hospital of Padua, Padua, Italy; 7 Department of Surgery, University Hospital of Verona, Verona, Italy; 8 Upper GI Surgery Unit, Humanitas Research Hospital, Milan, Italy; 9 Department of Molecular Medicine DMM, University of Padua, Padua, Italy

## Abstract

**Background:**

Ulcerative colitis patients and transplant recipients are at risk for colorectal cancer. Here, we show that immunosuppressive regimens for kidney transplants are associated with the progression of ulcerative colitis-related carcinogenesis.

**Methods:**

We describe the case of a patient diagnosed with colorectal cancer in ulcerative colitis while on immunosuppressive therapy for a kidney transplant. The immunological microenvironment of the cancer and its mutational status were analyzed, and a mouse colon cancer model was created to replicate the unique clinical conditions. AOM/DSS mice were randomized into seven experimental groups that received different immunosuppressants and an untreated control group to assess the frequencies of adenocarcinoma and high-grade dysplasia. Histopathology, immunohistochemistry, and flow cytometry were also performed on the harvested mouse colons.

**Results:**

All mice treated with an immunosuppressive regimen developed at least an adenoma, and several of those receiving anti-CD3, anti-CD8, and mycophenolate mofetil also developed adenocarcinomas. In contrast, mice receiving rapamycin did not develop adenocarcinomas, and the extent of high-grade dysplasia in those mice was similar to that in control mice.

**Conclusions:**

Patients with pre-neoplastic conditions, such as ulcerative colitis, who are undergoing a solid organ transplant might benefit from the use of mTOR inhibitors given their intrinsic anti-tumor properties.

## Abstract (web summary)

Among transplant recipients, colorectal cancer is more aggressive. This report highlights the association between immunosuppression and the disruption of the immune surveillance mechanisms against colorectal cancer.

## Introduction

The association between inflammation and the risk of colorectal cancer (CRC) is well documented in animal models and in humans, but the interplay between acquired immunity (and its pharmacologic suppression) and CRC progression in inflammatory carcinogenesis is less well understood. The tumor microenvironment includes a complex network of T cell subpopulations that directly interact with cancer cells and ultimately influence the clinical course and that are the foundation of a more general process of cancer immunoediting^1^. High expression levels of the cytotoxic and Th1 clusters within CRC are associated with prolonged disease-free survival, suggesting that these subpopulations might play an active role in cancer immune editing^2–4^. Successful tumor protection occurs after immunization in mice depleted of CD4(+) but not CD8(+) T cells, suggesting that tumor protection is largely CD8-mediated and CD4-independent^5^. Therefore, it may be hypothesized that immunosuppression has an enhancing effect on CRC progression. In fact, C57BL/6-Apc(Min/+) mice, a model for human colon cancer, depleted of CD4(+) and CD8(+) lymphocytes developed twice as many tumors as immunocompetent mice^6^. However, the role of immunosuppression is more difficult to predict because metastases of CT26 were decreased in CD4+ T cell-depleted BALB/c mice, suggesting that CD4+ T cells are involved in negative regulation of anti-tumor responses^7^. In fact, in DSS-AOM-treated mice, transient ablation of CD4/Foxp3 Treg, during the carcinogenesis, suppressed tumor outgrowth and distribution, accompanied by an increased number of CD8 effector T cells^8^. Moreover, in an inflammatory mouse model, using a standard sequential exposure to AOM followed by DSS treatment, the tumor incidence in WT mice was 58%, while TCR-deficient mice showed lower adenoma incidences, and none of the immunocompromised mice developed adenocarcinomas^9^. Finally, in a mouse model of colon adenocarcinoma, the depletion of CD4+CD25+regulatory T cells with anti-CD25 antibodies enhances interleukin-2-induced anti-tumor immunity^10^.

An increased risk of CRC has been observed among solid organ transplant recipients relative to the general population, with standardized incidence ratios (SIR) ranging from no association up to a two-fold increase^11,12^, an overall SIR estimate of 1.69 reported in a meta-analysis^13^, and an overall SIR estimate of 1.24 (1.15–1.34) reported in a broad population-based study^14^. Among the transplant recipient population, proximal colon cancer risk is increased by the presence of underlying medical conditions and specific immunosuppressive regimens^15^. Moreover, these patients are often younger at diagnosis than those in the general population, and their 5-year survival rate is also significantly lower than for other patients with CRC^16^. This worse prognosis is most likely related to increased tumor aggressiveness, reduced immunological response, or both^17^.

Here, we describe a young man with an aggressive adenocarcinoma of the anal transitional zone arising after restorative proctocolectomy for a previous early rectal neoplasia in UC. The patient had received a kidney transplant after colon removal and thus had undergone multiple immunosuppressive therapies, including cyclosporine A, tacrolimus, mycophenolate mofetil, prednisone, and anti-thymocyte globulins. We describe the association between these immunosuppressive medications and the disruption of the immune surveillance mechanisms against inflammation-related CRC.

## Case report

**Fig. 1:**
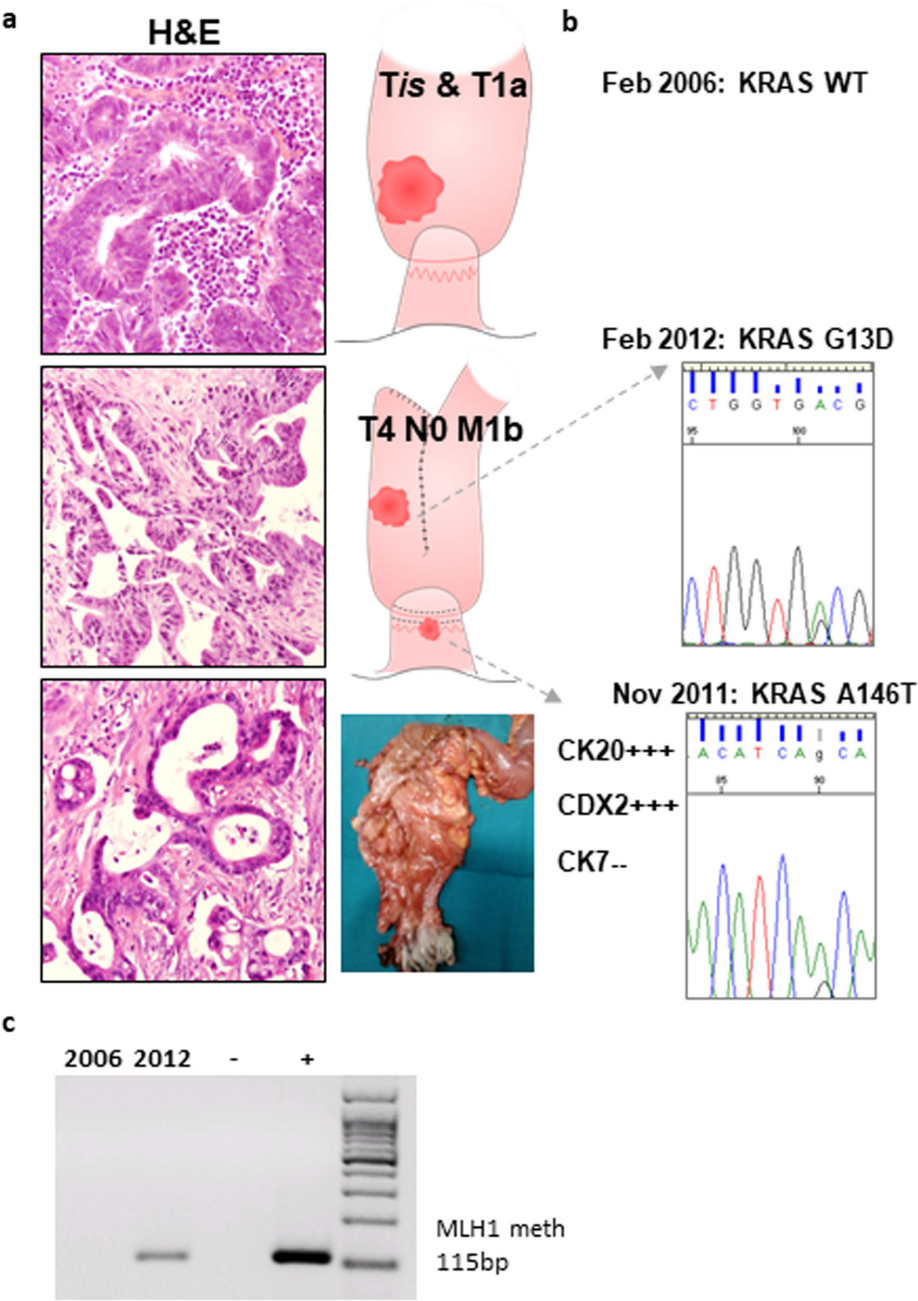
Characterization of tumor specimens. **a** Representative histological features of the different lesions observed in the case reported (hematoxylin and eosin staining). **b** Screening for mutations was performed on a Sequenom MassARRAY platform with the *Myriapod* Colon status and the *Myriapod* Lung status panels (Diatech Pharmacogenetics, Jesi, Italy). In each case, DNA was prepared after enrichment for neoplastic cellularity to at least 60%, using manual microdissection of eight consecutive 4-µm FFPE sections, and purified using the QIAamp DNA FFPE Tissue Kit (Qiagen). One-hundred and twenty nanograms of DNA were used for each multiplex PCR amplification, and the reactions were performed according to the manufacturer’s instructions. **c** Genomic DNA was extracted from the tissues using a DNeasy Blood & Tissue Kit (Qiagen), according to the manufacturer’s directions. Sodium bisulfate modification of gDNA was performed using the EZ DNA Methylation-Gold Kit (Zymo Research), following the manufacturer’s instructions. The primers for the *hMLH1* methylation-specific PCR and the PCR conditions are outlined in Supplementary Table 2. The EpiTect PCR Control DNA Set (Qiagen) was used as the positive control for the methylated and unmethylated MLH1 gene. The PCR products were resolved by gel electrophoresis, and each case was scored as methylated or unmethylated

We report the case of a patient who was diagnosed with ulcerative colitis (UC) in his third decade and who required several hospital admissions for UC flares, which were treated with sulfasalazine and steroids with good results. During one of these UC flares, chronic kidney failure of unknown origin was diagnosed. Thus, the patient received a living-donor kidney transplant and underwent immunosuppressive treatment. However, shortly after, because of a severe CMV infection refractory to antiviral treatment, it was necessary to remove the transplanted kidney. In the meanwhile, during the endoscopic follow-up, the patient was diagnosed with a dysplasia-associated lesion or mass (DALM) in the sigmoid colon and an early adenocarcinoma in the lower rectum. Therefore, a restorative proctocolectomy with J pouch and a stapled ileal pouch-anal anastomosis were performed. The pathologic stages were T1aN0M0 for the rectal carcinoma and high-grade dysplasia for the sigmoid DALM. Few years later, the patient underwent a kidney transplant from a cadaveric donor and, consequently, also underwent immunosuppressive therapy with cyclosporine-A, mycophenolate mofetil, and methyl prednisolone. A few months later, because of an episode of acute rejection, the patient had a short course of anti-thymocyte globulin and cyclosporine was shifted to tacrolimus. During the follow-up, the planned pouch endoscopy was delayed due to the occurrence of the acute rejection episode. Five months later, an increase in the level of CA 19.9 led to a PET scan and two areas of hyper-metabolism were found in the pouch (SUV = 9.9) and in the liver (SUV = 4.92). The pouch endoscopy showed a neoplasia at the ileo-anal anastomosis in the cuff region. The histology and the immunohistochemistry confirmed the diagnosis of colonic adenocarcinoma (CK20 and CDX2 positive, CK7 negative) of the anal transitional zone. Consequently, 2 months later, he was operated on, and during the laparotomy, a diffuse neoplasia of the pouch invading the pre-sacral fascia with peritoneal and liver metastasis was found. An abdomino-perineal pouch excision with end ileostomy was performed. The pathology report confirmed the diagnosis of mucinous adenocarcinoma (T4N1M1). Immunosuppression was minimized, and although the introduction of mTOR inhibitors was planned, they were never started because of the poor general condition of the patient. Despite chemotherapy with bevacizumab-FOLFOX4, the patient died 10 months after diagnosis. The patient (and all the control patients with different type of CRC) provided written informed consent to have his data used for scientific purposes. Moreover, the patient (and all the control patients with different type of CRC) participated in the MICCE1 study approved by the Institutional Review Board (IRB) at the Padua University Hospital. All biopsies and molecular testing were performed in accordance with protocols approved by the same IRB. The histology and cancer site of the three different tumors are shown in Fig. 1a.

## Results

### Genetic analysis and cellular characterization of tumor specimens

To study the genetic drivers of the patient’s tumors, we assessed the mutational status (Fig. 1b) and the methylation status of MLH1 gene (Fig. 1c) of the three different lesions. The screening for mutations on a Sequenom MassARRAY platform did not show any mutations in the 10 examined genes (*ALK*, *BRAF*, *DDR2*, *EGFR*, *ERBB2*, *KRAS*, *MAP2K1*, *NRAS*, *PIK3CA*, *RET*) in the DALM or in the first adenocarcinoma removed a few years before the kidney transplant. The second lesion, which developed on the anal transitional zone and was biopsied at diagnosis, was characterized by an A147T mutation in exon 4 of the *KRAS* gene, while a G13D mutation in exon 2 of the *KRAS* gene was observed in the third lesion removed 2 months later.

### Immune microenvironment of tumor specimens

**Fig. 2:**
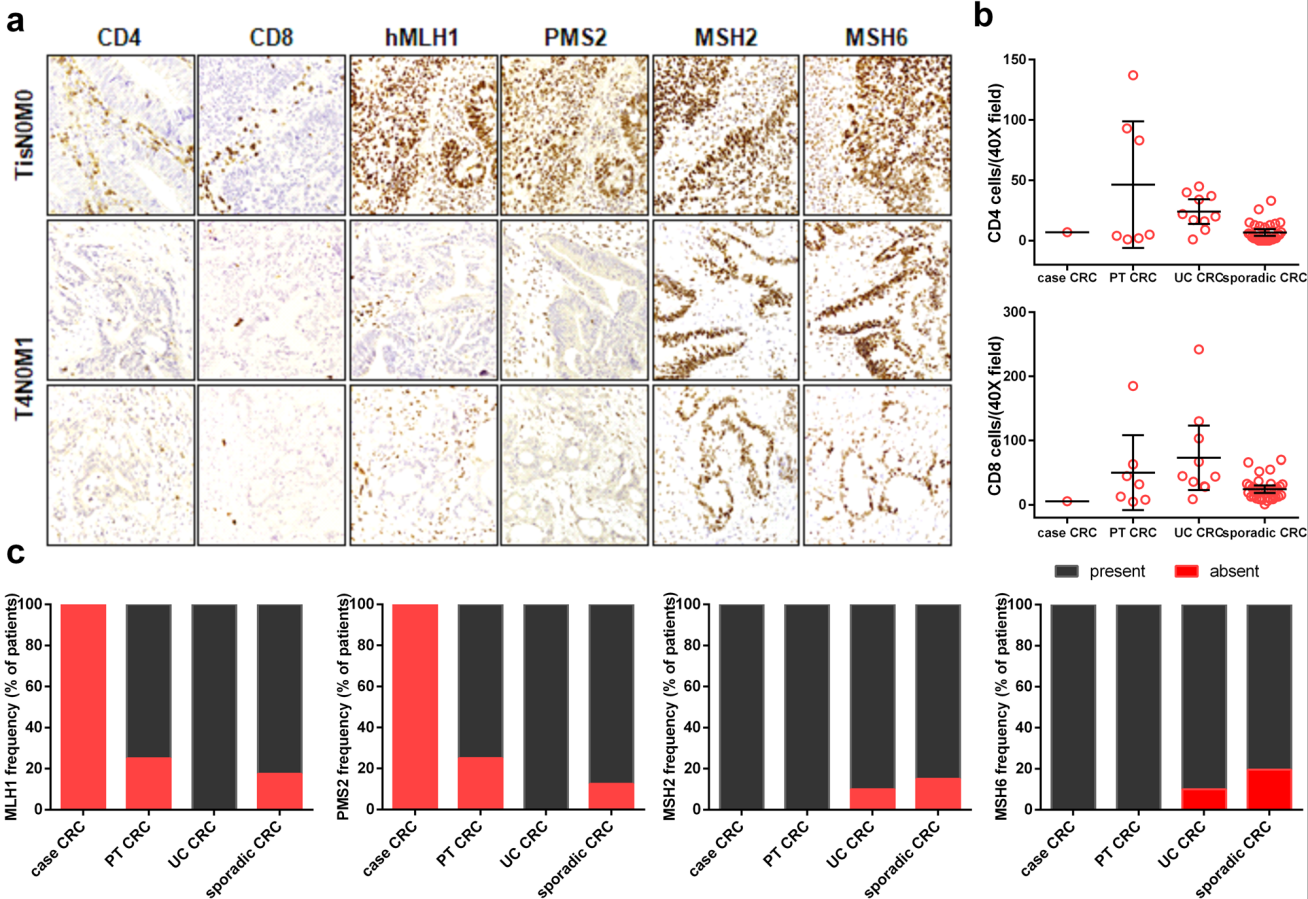
Post-transplant immunosuppression and enhanced immune response in UC-related carcinogenesis. **a** Immunohistochemical analyses were performed using standard procedures, and the resulting sections were evaluated by a single pathologist in a blinded fashion. Immunocomplexes were detected using the Dako Real Envision System Peroxidase/DAB (Dako, Glostrup, Denmark) in sections from formalin-fixed and paraffin-embedded specimens. The antibodies used for immunohistochemistry are shown in Supplementary Table 3. Representative images from our patient are shown. **b** We retrospectively enrolled post-transplant CRC patients (PT CRC, *n* = 8 patients), UC-related CRC patients (UC CRC, *n* = 10 patients), and sporadic CRC patients (*n* = 42 patients) and compared their 95% CIs of CD4 and CD8 infiltration to those of our patient, who had post-transplant UC-related CRC (case CRC, *n* = 1). The expression levels of CD4 and CD8 were measured quantitatively (they were manually counted at 40× magnification in 10 random fields), whereas the expression of the mismatch repair proteins was classified as present or absent (**c**)

To study the immune microenvironment of the patient’s tumors, we assessed the infiltration of the CD4+ and CD8+ T lymphocytes and the expression of the mismatch repair genes (MMR) of the three different lesions (Fig. 2). The immune microenvironment showed an unexpected evolution. In the DALM and the T1a adenocarcinoma removed a few years before the kidney transplant, CD4+ and CD8+ T lymphocytes had evenly infiltrated the neoplasms. In contrast, in the adenocarcinoma of the anal transitional zone and the adenocarcinoma removed 2 months later, both T cell subpopulations were dramatically reduced (Fig. 2a).

To analyze the double effect of the post-transplant immunosuppression and the immune-enhancing UC-related carcinogenesis in humans, we enrolled 8 patients with post-transplant CRC, 10 patients with UC-related CRC, and 42 patients with sporadic CRC, and we compared them with our patient. Patient characteristics are shown in Supplementary Table 1. In our patient, CD4 infiltration within the tumor was similar (within the 95% CI) to that observed in post-transplant CRC and in sporadic CRC, while that observed in UC-related CRC was significantly higher. Moreover, in our patient, CD8 infiltration was similar to that in post-transplant CRC patients, not that in UC-related CRC patients, as expected (Fig. 2b).

In our patient, the immunohistochemical profile of the MMR proteins was consistent with microsatellite stability in both the DALM and the first adenocarcinoma, whereas both MLH1 expression and PMS2 expression were negative in the subsequent two tumors (Fig. 2a). Methylation-specific PCR showed that the MLH1 promoter was methylated in the last adenocarcinoma, while it was not in the one before the kidney transplant (Fig. 1c). Similarly, a high frequency of the lack of hMLH1 and PMS2 expression was observed in post-transplant CRC patients, including our patient. In contrast, this MMR gene expression defect was not observed in UC-related CRC patients (Fig. 2c).

### Effects of different immunosuppressants on a murine inflammatory colon carcinogenesis model

**Fig. 3:**
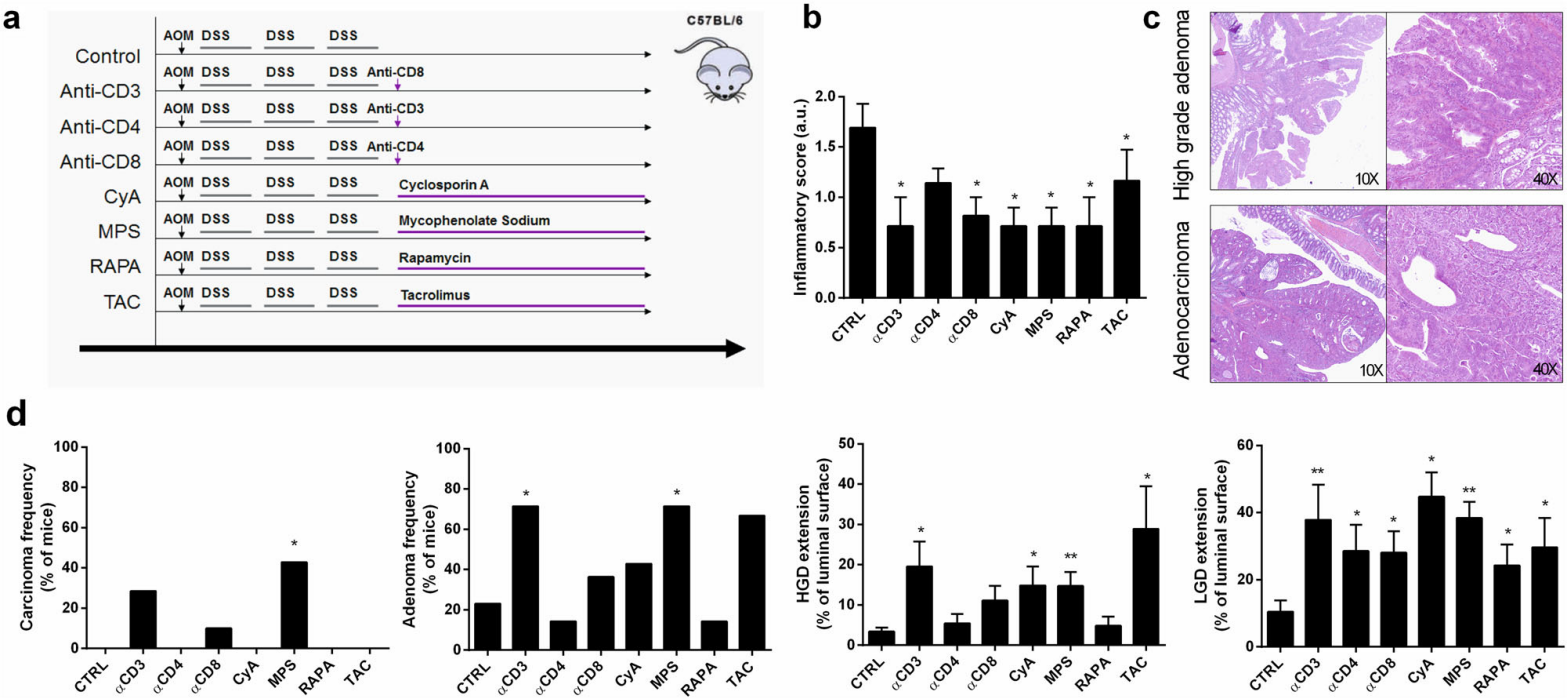
Effects of different immunosuppressants on a murine inflammatory colon carcinogenesis model. **a** Animal experiments were performed according to Italian Law 116/92 and European directive 2010/63/UE. Experimental protocols were reviewed and approved by the Institutional Animal Care and Use Committee (Comitato Etico Scientifico per la Sperimentazione Animale) of the University of Padova, Padova, Italy. The experimental design of the mouse model of inflammation-driven colon carcinogenesis involved cohoused 12-wk-old C57Bl6/J male mice that were injected with azoxymethane (AOM, Sigma) intraperitoneally (i.p.) at a dose of 7.4 mg/kg body weight (mean weight 27 ± 1 g). After 4 weeks, the mice received 2.5% dextran sodium sulfate (DSS, Applichem) (M.W. 40,000 g/mol) in their drinking water for 5 days, followed by 2 weeks of plain water. This cycle was repeated two more times. One week after the third DSS treatment, the AOM/DSS mice were randomized (randomization based on a single sequence of computer-generated random numbers) into seven experimental groups receiving immunosuppression and an untreated control group (*n* = 10). All the mice alive at the end of the experiment were included in the study. Three groups were treated i.p. with 200 μg/mouse of anti-CD3 Ab (clone 145-2C11, ATCC hybridoma no. CRL-1975) (*n* = 7), anti-CD4 Ab (clone GK1.5, ATCC hybridoma no. TIB-207) (*n* = 7), or anti-CD8 (clone 2.43, ATCC hybridoma no. TIB-210) (*n* = 10) monthly. Mice were euthanized 4 weeks after the antibody injection for tissue collection. Cyclosporine A (10 mg/kg/day, CyA) (Sandimmun, Novartis) (*n* = 7), tacrolimus (1 mg/kg/day, TAC) (Prograf, Astellas) (*n* = 7), mycophenolate-mofetil sodium (30 mg/kg/day, MPS) (Myfortic, Novartis) (*n* = 7), or rapamycin (1 mg/kg/day, RAPA) (Rapamune, Pfizer) (*n* = 7) were administered by oral gavage (200 µl) for 4 consecutive weeks to the additional four AOM/DSS groups. The dosages were prepared according to an average weight of the experimental mice of 27 g. All mice were sacrificed 24 h after the last oral gavage for tissue collection. Sections (4 μm) from formalin-fixed and paraffin-embedded specimens were stained with hematoxylin-eosin and evaluated by a single pathologist in a blinded fashion. **b** Histological inflammation and dysplasia presence and extent were quantified and classified by a pathologist (S.M.) unaware of the arm of the experiment using Floren’s score^26^ and the Vienna classification of gastrointestinal epithelial neoplasia^27^. **c** Representative histopathological lesions observed in the inflammation-driven colon carcinogenesis model. **d** Murine colons were analyzed for dysplasia at high magnification (40×). The extent of dysplasia was quantified as the percentage of total bowel length represented by the length of the involved bowel. Carcinoma and adenoma frequencies were macroscopically quantified over the whole length of each mouse colon and then verified with histology. Each treatment group was compared to control mice who were treated only with AOM and DSS. Fisher’s exact test and the Mann–Whitney U test were used to compare dichotomous and continuous variables, respectively (*p < 0.05, **p < 0.01)

To verify the role of the immunosuppressive therapy in accelerating the progression of colonic carcinogenesis, we tested the medication that the patient received or would have received on a mouse model of inflammation-driven colon carcinogenesis (Fig. 3a). Inflammation was significantly inhibited in AOM/DSS mice treated with anti-CD3, anti-CD8, or immunosuppressant drugs (CyA, MPS, TAC, and RAPA) compared to the inflammation in control mice that received only AOM/DSS (Fig. 3b). All the mice that received immunosuppressive drugs showed significantly more extensive low-grade dysplasia (LGD) than control mice (*p* = 0.01). However, the extent of high-grade dysplasia (HGD) was greater in mice that received anti-CD3 ab, CyA, MPS, or TAC than in control mice or in those that received anti-CD4 ab or RAPA (*p* = 0.01). HGD and adenocarcinoma histology is exemplified in Fig. 3c. Moreover, only mice that received anti-CD3 ab, anti-CD8 ab, and MPS (*p* = 0.05) developed adenocarcinomas after 4 weeks of therapy, while all the other groups only developed adenomas (Fig. 3d).

### Effects of different immunosuppressants on mucosal immune microenvironment

**Fig. 4:**
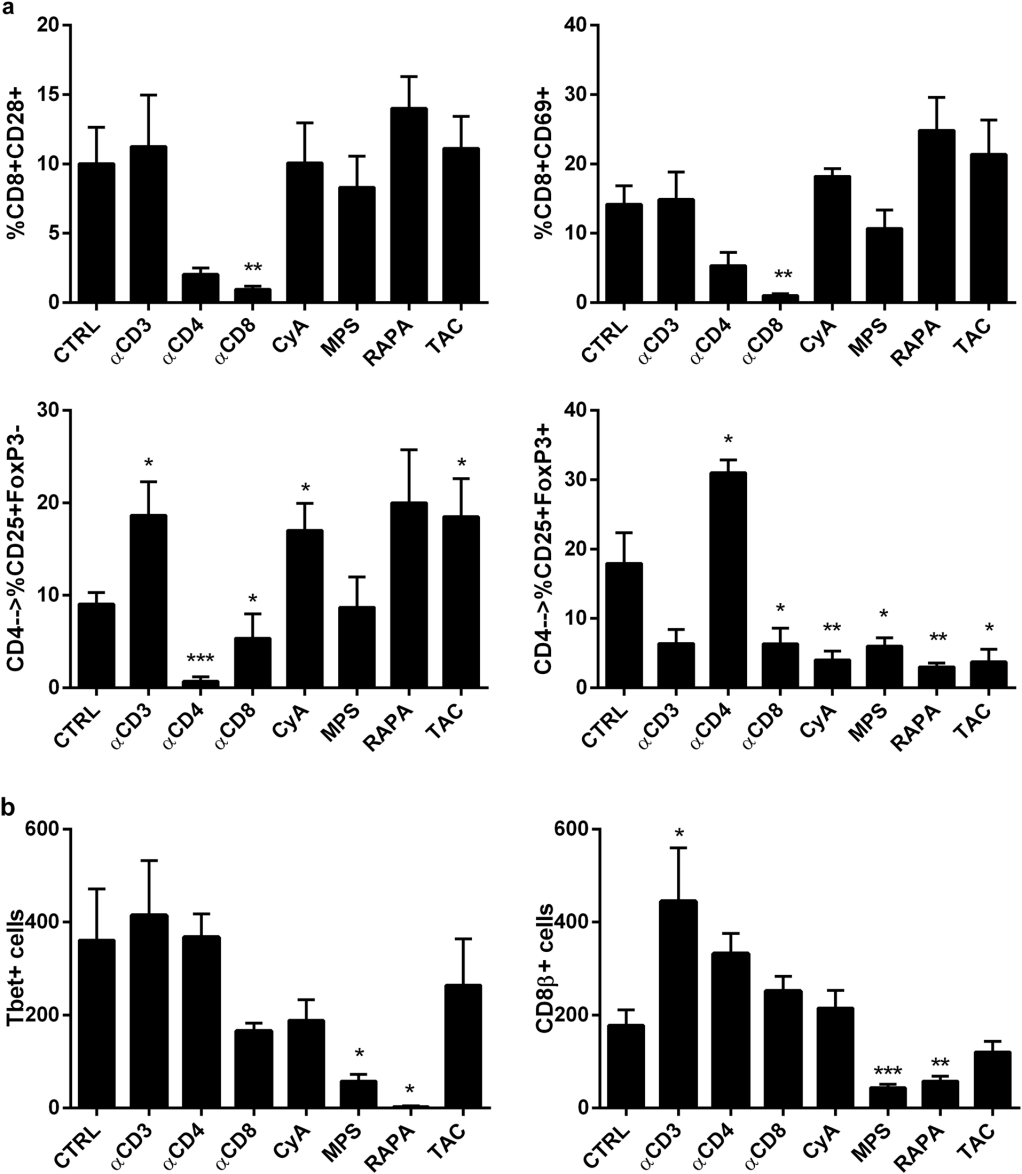
Effects of different immunosuppressants on the mucosal immune microenvironment. **a** Mouse colonic samples were incubated in HBSS supplemented with 1 mM DTT and 0.5 mM EDTA with shaking at 37 °C for 20 min. After washing, the tissues were treated with 1 U/ml dispase (Stemcell Technologies) in DMEM containing 5% FCS at 37 °C for 30 min with gentle stirring. Single-cell suspensions were subjected to flow cytometry to determine the proportion of activated CD8+ T cells (positive for CD28 and CD69) and CD4+ T cells (CD25+FoxP3− and CD25+FoxP3+). Flow cytometric analysis was performed using a FACSCalibur based on CellQuest software (Becton Dickinson). The antibodies used are summarized in Supplementary Table 4. **b** Immunohistochemical analyses were performed using standard procedures, and the resulting sections were evaluated by a single pathologist in a blinded fashion. The antibodies used for immunohistochemistry are shown in Supplementary Table 3. Cells expressing CD8β and Tbet were manually counted at 40× magnification in 10 random fields from consecutive slides (*n* = 6 for each treatment group). Each treatment group was compared to control mice who were treated only with AOM and DSS. Statistical significance was assessed by Mann–Whitney U tests (*p < 0.05, **p < 0.01, ***p < 0.001)

The proportions of activated naive cytotoxic T lymphocytes (CD8+CD69+) and those expressing the CD80 receptor (CD8+CD28+) in the colon of AOM-DSS mice were decreased only by anti-CD8 treatment and were not affected by the immunosuppressive medications (Fig. 4a). In contrast, the proportion of activated cytotoxic lymphocytes (CD8beta+) was significantly lower in the MPS (*p* = 0.0007) and RAPA groups (*p* = 0.004) and higher in the anti-CD3 group (*p* = 0.04) (Fig. 4b) than in the other groups.

Activated T-helpers (CD4+CD25+FoxP3−) were significantly reduced in mice receiving anti-CD4 and anti-CD8 treatment compared to control mice (Fig. 4a). In contrast, the T-reg (CD4+CD25+FoxP3+) subpopulation was significantly reduced in mice receiving anti-CD8, CyA, TAC, MPS, and RAPA compared to control mice. In contrast, in mice receiving anti-CD4, the levels of T-reg cells were increased compared to those in the control mice. Moreover, numbers of Tbet+ cells were significantly reduced in mice receiving RAPA compared to control mice (*p* = 0.025) (Fig. 4b). As shown in Supplementary Fig. 1, in the non-dysplastic colons of mice treated with AOM-DSS, the Tbet/CD4 ratio was well below 1, suggesting that all the Tbet+ cells were CD4+ and thus Th1, independent of whether the mice were immunosuppressed or not. In contrast, within the dysplastic lesions in non-immunosuppressed mice, the Tbet/CD4 ratio was well above 1, suggesting that a portion of the Tbet+ cells were also CD8+, while in immunosuppressed mice the ratio fell back below 1. Tacrolimus seemed to be an exception. However, Tbet and CD4 staining was performed on independent sections, so we used Tbet/CD4 ratio as surrogate measure, and we cannot exactly determine how much the observed effects on Tbet expression reflects an impact on CD8 or CD4 T cells.

The analysis of the epithelial cells acting as antigen-presenting cells showed a significant increase of epithelial cells expressing CD80 in mice receiving MPS and in those receiving RAPA (*p* = 0.01), and a significant increase of epithelial cells expressing MHC-I in mice receiving MPS (*p* = 0.01) compared to control mice (Supplementary Fig. 2).

## Discussion

This patient presented with an advanced adenocarcinoma of the anal transitional zone 1 year after a kidney transplant and 6 years after his first rectal cancer. The timing (6 years later), the stage at onset, the *KRAS* mutation pattern and the MMR gene status were different between the cancers, but the different speed of progression was the most striking, and fatal, feature. The influence of immunosuppressive regimens on malignant behavior in kidney recipients has long been debated^13–16^. In those patients on a TAC plus MPS plus prednisone-based regimen, the appearance of malignancies has been noted significantly earlier, suggesting a quicker cancer progression^18^. Interestingly, our patient received a massive immunosuppressive load with anti-thymocyte globulins and a switch to a TAC plus MPS plus prednisone-based regimen after the episode of acute rejection, which occurred 6 months before the diagnosis of metastatic anal transitional zone adenocarcinoma. Moreover, this cancer showed an unexpected reduced T cell infiltration, contrary to what is usually observed in CRC with MMR defects or in UC-related CRC^19,20^. In fact, in our patient, CD4 and CD8 infiltration within the tumor was similar to that observed in post-transplant CRCs and lower than that observed in UC-related CRCs, suggesting a deep effect on cancer microenvironment of post-transplant immunosuppression regimen. Thus, our hypothesis is that the immunosuppressive medication caused the dramatic acceleration of the colonic inflammatory carcinogenesis.

We recently reported that in inflammatory colorectal carcinogenesis, the interplay between epithelial cells acting as antigen-presenting cells and cytotoxic lymphocytes through CD80-CD28 signaling controls the progression from LGD to HGD and then to cancer^21^. Our murine model was designed to analyze the early stages of inflammation-driven colon carcinogenesis, with a lower dose of AOM than is commonly used. Immunosuppression itself may directly interfere with the process of carcinogenesis^8^, and thus the adenoma rate was relatively low. Nevertheless, several of the mice receiving anti-CD3, anti-CD8, and MPS also developed adenocarcinomas. These results suggest that interfering with the number and function of the T lymphocytes might inhibit cancer initiation in chronic inflammation but that once cancer initiation starts, that interference might rapidly accelerate the cancer progression.

The T cell subpopulation analysis sheds light on the roles of different medications. Cytotoxic T cells seemed to be influenced only by anti-CD8 treatment, although the CD8beta expression of cytotoxic T cells was influenced by MPS and RAPA. On the other hand, T-reg cells seemed to be affected by anti-CD8, MPS, RAPA, CyA, and TAC. Therefore, in mice receiving MPS, the high T-reg cell/activated T-helper and low T-reg cell/cytotoxic T cell ratios showed a strong immune surveillance imbalance that might lead to tumor escape. In contrast, in mice receiving CyA, TAC, and RAPA, the T-reg cell subpopulation was reduced in the colonic mucosa, likely reducing their pro-tumorigenic effect^22^. These data seemed to explain the unexpected and significant rate of adenocarcinoma progression after only 1 month of immunosuppression with MPS. MPS is a morpholinoethyl ester of mycophenolic acid that has been extensively studied in several clinical trials, and it has shown a more favorable toxicity profile than azathioprine^23^. MPS has been shown to inhibit tumor cell growth and angiogenesis strongly in vitro but only marginally in vivo^24^. These contrasting results may relate to drug availability, as suggested by Koehl et al., but also to the unbalanced immunosuppression that we observed in this study. On the other hand, mTOR inhibitors might be a valid alternative. In fact, mice receiving RAPA did not develop adenocarcinomas, and the extent of HGD was similar to that in control mice. Indeed, RAPA, besides being an effective immunosuppressor, can cause a direct anti-tumor effect through the inhibition of mTOR, a serine/threonine kinase that controls cell size and proliferation and that is commonly deregulated in human cancers^25^. In our opinion, this medication should considered for patients who have to undergo solid organ transplant and have a high oncological risk.

In conclusion, our results highlight the risk of post-transplant cancer in patients with precancerous conditions. Paradoxically, despite the complete removal of the colon and the rectum, patients after restorative proctocolectomy are still at high risk if they had a previous colonic dysplasia or cancer. In these cases, post-transplant endoscopic surveillance should be conducted regularly. In fact, the immune impairment due to immunosuppressive medication can be associated with the progression of colonic cancer. MPS seems to be more active than other drugs in accelerating carcinogenesis, while mTOR inhibitors might be a safer alternative given its intrinsic anti-tumor properties.

## Electronic supplementary material

Supplementary material and methods

Supplementary figure 1

Supplementary figure 2
