# Correction: Effects of immune suppression for transplantation on inflammatory colorectal cancer progression

**DOI:** 10.1038/s41389-018-0068-0

**Published:** 2018-07-31

**Authors:** Imerio Angriman, Lucrezia Furian, Melania Scarpa, Matteo Fassan, Susan Morgan, Andrea Porzionato, Andromachi Kotsafti, Luca Saadeh, Cristina Silvestre, Raffaele De Caro, Amedeo Carraro, Umberto Tedeschi, Romeo Bardini, Paolo Rigotti, Massimo Rugge, Carlo Castoro, Ignazio Castagliuolo, Marco Scarpa

**Affiliations:** 1 General Surgery Unit, University Hospital of Padua, Padua, Italy; 2 Kidney Transplant Unit, University Hospital of Padua, Padua, Italy; 3 Esophageal and Digestive Tract Surgical Unit, Veneto Institute of Oncology IOV-IRCCS, Padua, Italy; 4 Surgical Pathology Unit, Department of Medicine DIMED, University Hospital of Padua, Padua, Italy; 5 Pathology Unit, Sheffield Teaching Hospitals, Sheffield, UK; 6 Department of Neurosciences, University Hospital of Padua, Padua, Italy; 7 Department of Surgery, University Hospital of Verona, Verona, Italy; 8 Upper GI Surgery Unit, Humanitas Research Hospital, Milan, Italy; 9 Department of Molecular Medicine DMM, University of Padua, Padua, Italy

Correction to**:** *Oncogenesis* 10.1038/s41389-018-0055-5; published online 19 june 2018

At the time of publication, the html version of this paper contained an error; the authors Imerio Angriman and Lucrezia Furian were not tagged as equally contributing authors. This has now been fixed in the html version of the paper, the PDF was correct at the time of publication.

