# Supplementary material for: Effects of immune suppression for transplantation on inflammatory colorectal cancer progression
Source: Oncogenesis. 2018 Jun 19;7(6):46. doi: 10.1038/s41389-018-0055-5 (PMC6006312; doi:10.1038/s41389-018-0055-5)
Supplement: Supplementary file 1 — Supplementary material and methods [file 41389_2018_55_MOESM1_ESM.docx]

**Supplementary Methods**

*Mutational analysis*

In each case, DNA was prepared after enrichment for neoplastic cellularity to at least 60% using manual microdissection of 8 consecutive 4-µm FFPE sections and purified using the QIAamp DNA FFPE Tissue Kit (Qiagen). One-hundred and twenty nanograms of DNA were used for each multiplex PCR amplification, and reactions were performed according to manufacturer’s instructions.

*Histopathology*

Sections (4 μm) from formalin-fixed and paraffin-embedded human and mice specimens were stained with haematoxylin-eosin. Histological inflammation was quantified and classified by a pathologist (S.M.) unaware of the arm of the experiment using Floren's score [1] and the Vienna classification of gastrointestinal epithelial neoplasia [2]. Murine colons were analyzed for dysplasia at high magnification (40x). The extent of dysplasia was quantified as the percentage of involved bowel length. Inflammation was scored from 0 to 4, with normal colonic mucosa = 0, shortening and loss of the basal one-third of the crypts with mild inflammation in the mucosa = 1, loss of the basal two-thirds of the crypts with moderate inflammation in the mucosa = 2, loss of the entire crypts but not the surface epithelium with severe inflammation in the mucosa = 3, and loss of both entire crypts and surface epithelium with severe inflammation in the mucosa = 4 [3].

*Immunohistochemistry*

Immunohistochemical analyses were performed using standard procedures, and the resulting sections were evaluated by a single pathologist in a blinded fashion. Immunocomplexes were detected using the Dako Real Envision System Peroxidase/DAB (Dako, Glostrup, Denmark) in sections, from formalin fixed and paraffin embedded human and mice specimens. In human tissues, the expression of CD4, CD8 was measured semi-quantitatively: none (0), mild (1-15), moderate (16-30) and severe lymphocytic infiltration (˃ 30), whereas, mismatch repair proteins expression was classified as present or absent. In mice, cells expressing CD8β and Tbet were manually counted at 40x magnification in 10 random fields. The antibodies used for immunohistochemistry are shown in Supplementary Table 1.

*Methylation specific PCR*

Genomic DNA was extracted from tissues using a DNeasy Blood & Tissue Kit (Qiagen) according to the manufacturer’s directions. Sodium bisulfate modification of gDNA was performed using the EZ DNA Methylation-Gold Kit (Zymo Research) following the manufacturer’s instructions. The primers for MLH1 methylation-specific PCR and PCR conditions are outlined in Supplementary Table 3. The EpiTect PCR Control DNA Set (Qiagen) was used as the positive control for the methylated and unmethylated MLH1 gene. PCR products were resolved by gel electrophoresis and each case was scored as methylated or unmethylated. The primers for MLH1 methylation-specific PCR and PCR conditions are outlined in Supplementary Table 2.

*Flow cytometry*

Mice colonic samples were freed of mucus by a 30-min wash in HBSS containing 10 mM DTT (Applichem). Then the mucosa was removed from the underlying muscle layer, and digested with 1 mg/ml collagenase and DNase (Sigma Aldrich) for 30 min at 37°C. Preparations were then preferentially enriched for LPMC using a Ficoll-Hypaque Plus gradient (GE Healthcare).

Mice colonic samples were incubated in HBSS supplemented with 1 mM DTT and 0.5 mM EDTA with shaking at 37 °C for 20 min. After washing, the tissues were treated with 1 U/ml dispase (Stemcell Technologies) in DMEM containing 5% FCS at 37 °C for 30 min with gentle stirring. Single-cell suspensions were subjected to flow cytometry to determine the proportion of epithelial cells (pan-cytokeratin+) acting as antigen-presenting cells (expressing CD80, CD40, MHC I or MHC II) and the proportion of activated CD8+ T cells (positive for CD28, CD38 or CD69) and CD4+ T cells (positive for CD25 and FoxP3). Flow cytometric analysis was performed using a FACSCalibur based on CellQuest software (Becton Dickinson). The antibodies used are summarized in Supplementary Table 3.

*Statistics*

Data are presented as the mean +/− SEM. The non-parametric Kruskall-Wallis ANOVA for multiple variables followed by Mann–Whitney's U-test for independent variables as post-hoc test were used for comparisons. The Kendall rank correlation test was performed. Differences were considered significant at p< 0.05.

The main outcome measure for our experiment was HGD and LGD extension. Considering an effect size of 50% and a standard deviation of 25% the subsequent standardized effect size was 2.0. Then we assumed a level of statistical significance (alpha) of 0.05 and a power (1-beta) of our tests of 0.20. Consequently, the minimal sample size required per group when using the two-tailed t test to compare means of continuous variables was at least of 6 mice per group. This number was chosen to minimize the number of mice necessary to complete the study.

**Supplementary references**

1. Florén CH, Benoni C, Willén R. Histologic and colonoscopic assessment of disease extension in ulcerative colitis. Scand J Gastroenterol. 1987;22:459–462.

2. Schlemper RJ, Riddell RH, Kato Y, Borchard F, Cooper HS, Dawsey SM, Dixon MF, Fenoglio-Preiser CM, Fléjou JF, Geboes K, Hattori T, Hirota T, et al. The Vienna classification of gastrointestinal epithelial neoplasia. Gut. 2000 Aug;47:251–5.

3. Zisman TL, Bronner MP, Rulyak S, Kowdley KV, Saunders M, Lee SD, Ko C, Kimmey MB, Stevens A, Maurer J, Brentnall TA. Prospective study of the progression of low-grade dysplasia in ulcerative colitis using current cancer surveillance guidelines. Inflamm Bowel Dis. 2012 Dec;18:2240–6.

**Supplementary Tables**

**Suppl. Table 1**: Antibodies used in immunohistochemistry

| **Antibody** | **Source** | **Company** | **Dilution** |
| --- | --- | --- | --- |
| Anti-human CD4 (clone 4B12) | Monoclonal mouse (M7310) | DakoDenmark A ⁄ S, Glostrup, Denmark | 1:40 |
| Anti-human CD8 (clone C8/144B) | Monoclonal mouse (M7103) | DakoDenmark A ⁄ S, Glostrup, Denmark | 1:50 |
| Anti-human LAMP-1 (H5G11) | Monoclonal mouse (sc-18821) | Santa Cruz Biotechnologies, California, USA | 1:50 |
| Anti-human β-catenin (E-5) | Monoclonal mouse (sc-7963) | Santa Cruz Biotechnologies, California, USA | 1:50 |
| Anti-human TLR4 [76B357.1] | Monoclonal mouse (ab22048) | Abcam Ltd., Cambridge UK | 1:100 |
| Anti-mouse CD8B | Rabbiti Polyclonal (orb1269) | Biorbyt Ltd., Cambridge, UK | 1:200 |
| Anti-mouse IL-17 | Rabbiti Polyclonal (13082-1-AP) | Proteintech Group, Inc. Illinois, USA | 1:200 |
| Anti-mouse TBX21 / T-bet | Rabbiti Polyclonal (LS-B10130) | LifeSpan BioSciences, Inc., Seattle, WA | 1:1500 |

**Suppl. Table 2**. Methylation specific PCR primers

| **Gene** | **sequence 5'-->3'** | **Ta,°C** | **Amplicon, bp** |
| --- | --- | --- | --- |
| MLH1  meth | Fw ACGTAGACGTTTTATTAGGGTCGC | 56 | 115 |
|  | Rv CCTCATCGTAACTACCCGCG |  |  |
| MLH1  unmeth | Fw TTTTGATGTAGATGTTTTATTAGGGTTGT | 56 | 124 |
|  | Rv ACCACCTCATCATAACTACCCACA |  |  |

**Suppl. Table 3**: Antibodies used in flow cytometry

| **Antibody** | **Company** |  |
| --- | --- | --- |
| Anti-mouse CD8a PE-Cy7 eBioscience Inc. | | |
| Anti-mouse CD28 FITC Abcam Ltd. | | |
| Anti-mouse CD38 FITC eBioscience Inc. | | |
| Anti-mouse CD69 PE Abcam Ltd. | | |
| Anti-mouse CD80 FITC eBioscience Inc. | | |
| Anti-mouse pan Cytokeratin PE Abcam Ltd. | | |
| Anti-mouse CD4 PE- Cy7 eBioscience Inc.  anti-mouse CD25 PE eBioscience Inc.  anti-mouse FoxP3 FITC eBioscience Inc. | | |

**Supplementary legends**

Supplementary Figure 1. A significant increase of epithelial cells expressing CD80 in mice receiving MPS and in those receiving RAPA and a significant increase of epithelial cells expressing MHC-I in mice receiving MPS were observed.
