# Supplementary figures and images for: Effects of immune suppression for transplantation on inflammatory colorectal cancer progression
Source: Oncogenesis. 2018 Jun 19;7(6):46. doi: 10.1038/s41389-018-0055-5 (PMC6006312; doi:10.1038/s41389-018-0055-5)

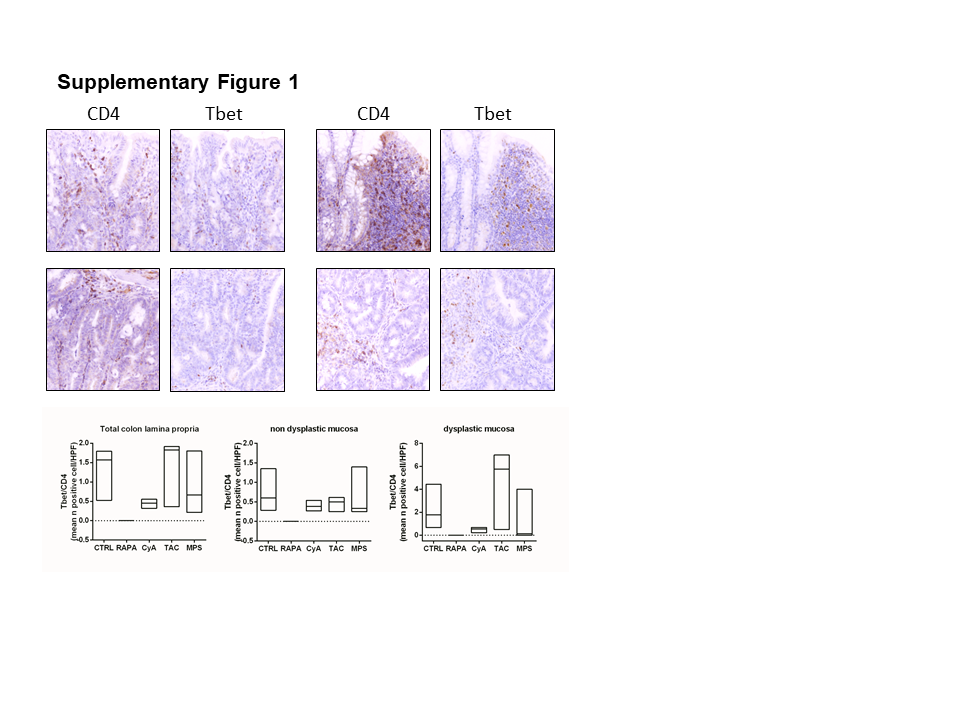

Supplement: Supplementary file 2 — Supplementary figure 1 [file 41389_2018_55_MOESM2_ESM.tif]

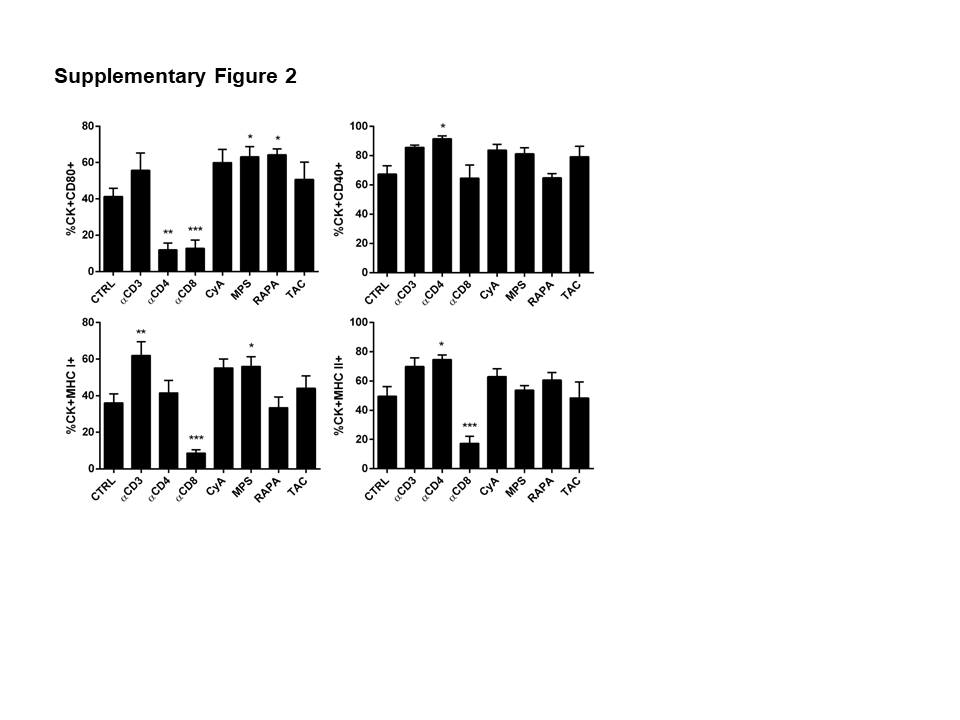

Supplement: Supplementary file 3 — Supplementary figure 2 [file 41389_2018_55_MOESM3_ESM.tif]
